# Modification of claims-based measures improves identification of comorbidities in non-elderly women undergoing mastectomy for breast cancer: a retrospective cohort study

**DOI:** 10.1186/s12913-016-1636-7

**Published:** 2016-08-16

**Authors:** Katelin B. Nickel, Anna E. Wallace, David K. Warren, Kelly E. Ball, Daniel Mines, Victoria J. Fraser, Margaret A. Olsen

**Affiliations:** 1 Division of Infectious Diseases, Department of Medicine, Washington University School of Medicine, 660 South Euclid Ave. Campus Box 8051, St. Louis, MO 63110 USA; 2 HealthCore, Inc., 123 Justison St Suite 200, Wilmington, DE 19801 USA; 3 Division of Public Health Sciences, Department of Surgery, Washington University School of Medicine, 660 South Euclid Ave. Campus Box 8100, St. Louis, MO 63110 USA

**Keywords:** Comorbidity, Administrative health claims data, Diabetes, Hypertension, Obesity, Mastectomy, Breast cancer

## Abstract

**Background:**

Accurate identification of underlying health conditions is important to fully adjust for confounders in studies using insurer claims data. Our objective was to evaluate the ability of four modifications to a standard claims-based measure to estimate the prevalence of select comorbid conditions compared with national prevalence estimates.

**Methods:**

In a cohort of 11,973 privately insured women aged 18–64 years with mastectomy from 1/04–12/11 in the HealthCore Integrated Research Database, we identified diabetes, hypertension, deficiency anemia, smoking, and obesity from inpatient and outpatient claims for the year prior to surgery using four different algorithms. The standard comorbidity measure was compared to revised algorithms which included outpatient medications for diabetes, hypertension and smoking; an expanded timeframe encompassing the mastectomy admission; and an adjusted time interval and number of required outpatient claims. A χ2 test of proportions was used to compare prevalence estimates for 5 conditions in the mastectomy population to national health survey datasets (Behavioral Risk Factor Surveillance System and the National Health and Nutrition Examination Survey). Medical record review was conducted for a sample of women to validate the identification of smoking and obesity.

**Results:**

Compared to the standard claims algorithm, use of the modified algorithms increased prevalence from 4.79 to 6.79 % for diabetes, 14.75 to 24.87 % for hypertension, 4.23 to 6.65 % for deficiency anemia, 1.78 to 12.87 % for smoking, and 1.14 to 6.31 % for obesity. The revised estimates were more similar, but not statistically equivalent, to nationally reported prevalence estimates. Medical record review revealed low sensitivity (17.86 %) to capture obesity in the claims, moderate negative predictive value (NPV, 71.78 %) and high specificity (99.15 %) and positive predictive value (PPV, 90.91 %); the claims algorithm for current smoking had relatively low sensitivity (62.50 %) and PPV (50.00 %), but high specificity (92.19 %) and NPV (95.16 %).

**Conclusions:**

Modifications to a standard comorbidity measure resulted in prevalence estimates that were closer to expected estimates for non-elderly women than the standard measure. Adjustment of the standard claims algorithm to identify underlying comorbid conditions should be considered depending on the specific conditions and the patient population studied.

**Electronic supplementary material:**

The online version of this article (doi:10.1186/s12913-016-1636-7) contains supplementary material, which is available to authorized users.

## Background

Adjusting for comorbidities in observational studies is essential to account for underlying differences in populations under investigation. This is especially true when studying healthcare utilization, costs, and patient outcomes, in which underlying comorbid conditions are associated with the outcomes. The Charlson index [1], a widely-used measure of 19 comorbidities, was adapted for administrative data using ICD-9-CM (International Classification of Diseases, 9^th^ Revision, Clinical Modification) codes [2, 3]. A second commonly used comorbidity measure with claims data developed by Elixhauser includes 29 medical conditions [4, 5]. These measures were developed and validated in hospitalized patients, and therefore may be more applicable to older, sicker populations.

A number of studies have compared the performance of the Charlson and Elixhauser measures [6–12]. The best measure has generally been determined based on improvement in explanatory power with regard to a specific outcome, usually mortality. Many studies have concluded that that these measures perform equally well [7, 9–11], while others have found the Elixhauser classification improved prediction of in-hospital [8, 12] and longer-term mortality [6, 12]. Several investigators have expanded the parameters of these two comorbidity measures to include physician [10, 13–15], outpatient, and auxiliary claims [10, 14, 15] and different look-back periods relative to the index event (e.g., one or two years of prior data and/or including the index admission) [10, 12, 14, 15].

We have an ongoing study of risk factors for postoperative complications in women under the age of 65 years after mastectomy and breast reconstruction using commercial insurer claims data [16, 17]. Breast reconstruction is most commonly performed in younger women [18] who have fewer underlying medical conditions (M. Olsen, unpublished results, National Inpatient Data) and lower annual healthcare costs than elderly women [19], suggesting less healthcare utilization. Fewer healthcare encounters and hospitalizations could limit the identification of comorbid conditions in claims data. For example, methods that perform well in elderly populations, e.g., using only diagnoses on inpatient hospital claims, may not be optimal for younger, generally healthy populations with infrequent hospitalizations.

To address some of the challenges of capturing comorbidities in younger populations, we selected diabetes, hypertension, anemia (including iron, folate, vitamin B12, protein, and other nutritional deficiency anemias), smoking, and obesity as representative examples of commonly occurring underlying health conditions in younger persons, and to illustrate the impact of modifications to the standard claims algorithm to improve capture of comorbidities. One alteration included the addition of diagnosis codes during the index hospital admission, to capture conditions that might be more commonly coded during a hospitalization than during prior outpatient encounters. All five conditions we included in our study pertain to this example, since they may be considered relevant to care provided during an inpatient hospitalization, but may not be relevant to service(s) or procedure(s) rendered during an outpatient visit, a requirement for health insurance coding [20]. Another alteration included allowing only a single diagnosis code to identify conditions for which testing is not necessary, such as obesity and smoking. A third alteration consisted of relaxing the requirement for spacing of diagnoses coded during outpatient encounters at least 30 days apart, because of the potential for clustering of care within a short time frame in younger women diagnosed with breast cancer. We also examined the effect of prescription drug claims to identify a condition, using hypertension and diabetes as relevant conditions. Our objective was to compare the impact of these modifications to the standard claims algorithm to identify a comorbid health condition, using the expected population prevalence of the comorbid health condition and medical record review to signal improvement.

## Methods

### Data source

We conducted a retrospective cohort study using the HealthCore Integrated Research Database (HIRD^SM^). We used data in the HIRD^SM^ from individuals enrolled in 12 Anthem-affiliated plans for this study. Anthem is an independent licensee of the Blue Cross and Blue Shield Association and serves its members as the Blue Cross licensee (California), and the Blue Cross and Blue Shield licensee (Colorado, Connecticut, Georgia, Indiana, Kentucky, Maine, Missouri (excluding 30 counties in the Kansas City area), Nevada, New Hampshire, New York, Ohio, Virginia (excluding the Northern Virginia suburbs of Washington, DC), and Wisconsin). Data in the HIRD^SM^ include all fully-adjudicated claims submitted for reimbursement from providers, facilities, and outpatient pharmacies linked to health plan enrollment information.

Fully insured women with health plan enrollment that included non-capitated medical coverage of hospital and physician services and prescription drug coverage were eligible for inclusion in the study cohort. Men were excluded due to the rare incidence of breast cancer in men. Women lacking continuous coverage from 365 days before through 30 days after mastectomy were excluded since comorbid conditions could not be fully measured. Additional exclusions included women with diagnosis codes for end-stage renal disease because of potentially incomplete claims, ICD-9-CM diagnosis code or prescription claim suggesting HIV positive status at any time for privacy considerations, and organ transplant in the year before mastectomy due to the rare nature of their underlying illness. Claims were restricted to paid claims.

The study data contained up to 5 ICD-9-CM diagnosis codes and 5 ICD-9-CM procedure codes per claim among women with a mastectomy in 2004–2008 and up to 12 ICD-9-CM diagnosis codes and 8 ICD-9-CM procedure codes among women with a mastectomy in 2009–2011. Inpatient facility claims also included Uniform Billing (UB-04) revenue and Healthcare Common Procedure Coding System (HCPCS) codes, while outpatient facility and provider claims included Current Procedural Terminology, 4^th^ edition (CPT-4) and HCPCS codes. Pharmacy claims contained National Drug Codes, which were linked to Generic Product Identifier codes to identify medications and drug groups.

### Patient population

We identified mastectomy operations among women aged 18–64 years from 1/1/2004–12/31/2011 using ICD-9-CM procedure codes 85.41–85.48 from inpatient facility claims and/or CPT-4 procedure codes 19180, 19200–19240, 19303, and 19305–19307 from outpatient facility and provider claims. We included only the first mastectomy per woman during the time period. The patient population was further refined by excluding operations coded by a provider- or facility-only without additional evidence for operation (i.e., anesthesia, pathology, or surgery revenue code) and mastectomy with codes for breast-conserving surgery if provider and other claims suggested breast-conserving surgery was more likely to increase the chance that a mastectomy was performed [17].

### Comorbidity algorithms

We used the ICD-9-CM diagnosis codes from the Elixhauser classification [4] to define diabetes, hypertension, anemia, and obesity (see Additional file 1). Since smoking is not included in the Elixhauser list of comorbidities; we used ICD-9-CM diagnosis codes for history of tobacco use and tobacco disorder to define smoking. We did not restrict the identification of conditions by diagnosis-related group [4] since we wanted to identify all relevant comorbid conditions at the time of mastectomy. We examined the impact of outpatient prescription claims for medications used to treat diabetes, hypertension, and smoking cessation to enhance the detection of these conditions (see Additional file 1). In order to focus on diagnoses recorded by clinicians, we excluded provider and outpatient facility claims containing only CPT-4 or UB-04 revenue codes for pharmacy, diagnostic radiology/cardiology/pulmonology, clinical laboratory, physical/occupational therapy, speech pathology or ambulance services. The timeframe used to identify comorbid conditions, frequency and interval between outpatient and provider claims, and use of prescription drug claims were changed sequentially to determine how the changes impacted the final prevalence calculated for each underlying condition (Table 1).

**Table 1:** Parameters for each algorithm used to identify comorbidities

Algorithm	Comorbidity	Timeframe relative to mastectomy date (in days)	Number of inpatient facility medical claims required	Interval and number of provider or outpatient facility medical claims required	Number of prescription drug (Rx) claims required
Algorithm 1	Diabetes, hypertension, deficiency anemia, smoking, obesity	−365 to −1	1+	2+ more than 30 days apart	n/a
Algorithm 2	Diabetes, hypertension, deficiency anemia, smoking, obesity	−365 to +7	1+	2+ more than 30 days apart	n/a
Algorithm 3	Diabetes, hypertension, deficiency anemia	−365 to +7	1+	2+	n/a
	Smoking, obesity	−365 to +7	1+	1+	n/a
Algorithm 4	Diabetes	−365 to +7 −365 to −1 Rx	1+	2+	1+
	Hypertension	−365 to +7 −365 to −1 Rx	1+	2+	1+ Rx plus 1 hypertension medical claim
	Smoking	−365 to +7 −365 to −1 Rx	1+	1+	1+

*n/a* not applicable

For the standard claims algorithm (Algorithm 1), we used medical claims from −365 through −1 days before mastectomy and required ≥ 2 provider or outpatient facility claims spaced > 30 days apart or ≥ 1 inpatient claim to identify comorbid conditions, as described by Klabunde [13]. For algorithm 2, we expanded the timeframe to capture comorbid conditions in medical claims through 7 days after mastectomy. While the standard claims algorithm described by Klabunde does not include identification of comorbid conditions during the hospital admission because some conditions could have new onset during the hospital admission (e.g., blood loss anemia, electrolyte disturbances), the comorbid conditions we selected for these analyses were most likely pre-existing at the time of mastectomy. Additionally, our study population consisted of younger, privately insured women with a low frequency of inpatient hospitalizations in the year prior to mastectomy, giving little opportunity to identify comorbid conditions in prior inpatient admissions. We suspected that some health conditions would be more likely to be coded during the mastectomy hospitalization (e.g., smoking) because of its relevance to surgery, so this provided additional motivation to determine the impact of adding the surgical hospitalization to the algorithm to identify underlying health conditions.

The frequency and interval requirements for provider/outpatient facility claims were altered in algorithm 3. Because of potential clustering of medical encounters in women with a diagnosis of breast cancer, we considered ≥ 2 provider/outpatient facility claims consistent with the diagnosis of interest (i.e., dropped the requirement for spacing of outpatient diagnoses > 30 days apart). For smoking and obesity, we considered single provider/outpatient facility claim(s) sufficient evidence since these conditions do not require diagnostic workup.

One or more prescription claims for medications to treat diabetes (oral hypoglycemic, insulin), hypertension, and smoking cessation from −365 to −1 days before mastectomy were added to algorithm 4 to detect these conditions (see Additional file 1). At least one medical claim with a hypertension diagnosis was required in addition to a prescription claim for an anti-hypertensive medication, since drugs used for hypertension may also be used to treat other conditions.

### Comparison populations

Survey results from the 2007 Behavioral Risk Factor Surveillance System (BRFSS) [21] and 2000 National Health and Nutrition Examination Survey (NHANES) [22] were used for the national estimates of the prevalence for each of the comorbid conditions. To best approximate our privately insured mastectomy cohort, weighted estimates for diabetes, hypertension, smoking, and obesity were calculated from female BRFSS respondents aged 18–64 years old with health insurance. In the surveys diabetes and hypertension were captured by the question “Have you ever been told by a doctor that you have (diabetes/high blood pressure)?” In both surveys BMI was defined using reported height and weight, and current smoking was defined on the basis of two questions: “Have you smoked at least 100 cigarettes in your entire life?” and “Do you now smoke cigarettes every day, some days, or not at all?” The prevalence of iron deficiency anemia (used as a proxy for all nutritional deficiency anemia) among adult females was available in a publication referencing NHANES data [22]. In the NHANES survey iron deficiency anemia was defined based on laboratory results from the respondent’s blood sample. The age group level prevalence estimates from the NHANES publication were averaged for 20–64 year olds to establish a single prevalence estimate for deficiency anemia. Because obesity is a risk factor for breast cancer [23], and hypertension and diabetes are more prevalent in obese persons, we assumed that women undergoing mastectomy would have higher rates of the selected conditions than the survey populations. While the survey populations may not be a gold-standard, the prevalence rates should serve as a baseline threshold for comparison to our study population.

For additional comparisons, a subset of the privately insured mastectomy population with postoperative ICD-9-CM diagnosis code(s) suggestive of wound complications was selected for medical record review. We selected this subset in order to validate the ICD-9-CM diagnosis codes for wound complications (manuscript in preparation), in addition to validation of the codes for obesity and smoking. For this subset height, weight, and current and past smoking history were abstracted from the medical records. Body mass index (BMI) ≥ 30 was used to define obesity.

### Analysis

Wald confidence intervals were calculated for each condition prevalence estimate. A chi-square test of proportions was used to compare the different algorithms in the mastectomy cohort and estimates from BRFSS and NHANES. Sensitivity, specificity, positive predictive value (PPV), and negative predictive value (NPV) were calculated to compare smoking and obesity data from the claims to the medical record results. All data management and statistical analyses were performed using SAS v9.3 (SAS Institute Inc., Cary, NC). This study was approved by the Human Research Protection Office at Washington University and by the Quorum Review IRB for the research activity at HealthCore.

## Results

The mastectomy cohort included 11,973 women aged 18–64 years with at least one year of health insurance enrollment prior to mastectomy from 1/1/2004–12/31/2011. The average age of women in the population was 51 years, and all 4 regions of the U.S. were represented in the cohort (Table 2). More than 60 % of women underwent mastectomy for locally invasive breast cancer, and 59 % of women had immediate reconstruction at the time of mastectomy, primarily involving an implant.

**Table 2:** Characteristics of 11,973 women in the mastectomy population

Characteristic	n (%)
Age, mean (standard deviation)	50.79 (8.36)
Region	
Northeast	2,122 (17.72)
South	3,966 (33.12)
Midwest	2,455 (20.50)
West	3,403 (28.42)
Unknown	27 (0.23)
Surgical procedure	
Mastectomy-only	4,887 (40.82)
Mastectomy with immediate implant reconstruction	5,440 (45.44)
Mastectomy with immediate flap reconstruction	1,228 (10.26)
Mastectomy with immediate flap plus implant reconstruction	418 (3.49)
Bilateral mastectomy	4,995 (41.72)
Indication for mastectomy	
Metastatic cancer	298 (2.49)
Regional cancer	2,385 (19.92)
Local breast cancer	7,381 (61.65)
Carcinoma *in situ*	1,529 (12.77)
Prophylactic	308 (2.57)
Benign/other	72 (0.60)
Inpatient operation	9,733 (81.29)

**Fig. 1:**
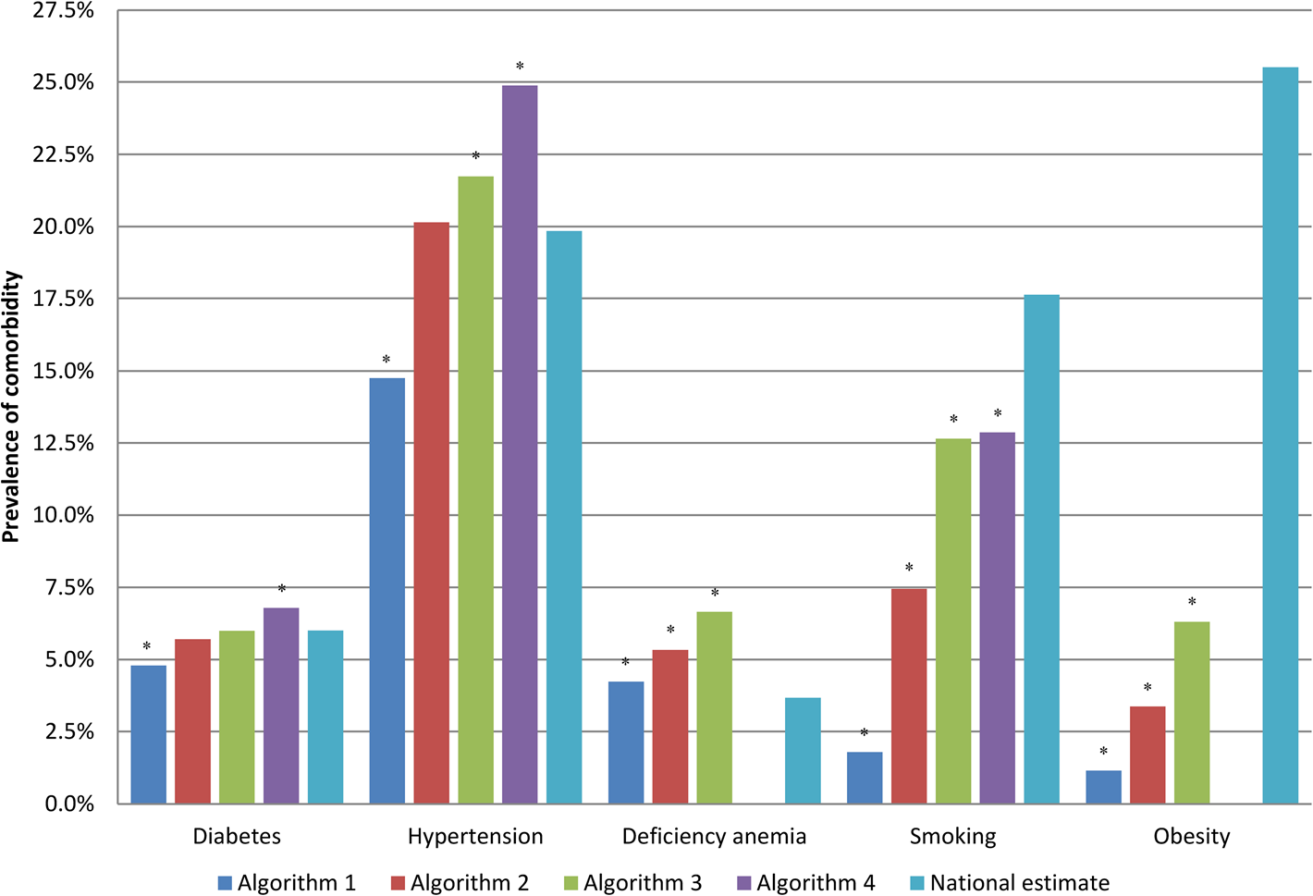
Comparison of Prevalence Estimates for Comorbidities by Algorithm Compared to National Estimates. ^*^ Significantly different from national estimate

For all five conditions, each successive change to the comorbidity algorithm resulted in an increase in prevalence of the condition (Fig. 1). Compared to the standard claims algorithm (algorithm 1), the revised prevalence estimates increased from 4.79 % (95 % confidence interval [CI] 4.40, 5.17) to 6.79 % (95 % CI 6.34, 7.24) for diabetes, 14.75 % (95 % CI 14.11, 15.39) to 24.87 % (95 % CI 24.10, 25.65) for hypertension, 4.23 % (95 % CI 3.87, 4.60) to 6.65 % (95 % CI 6.20, 7.09) for anemia, 1.78 % (95 % CI 1.54, 2.02) to 12.87 % (95 % CI 12.27, 13.47) for smoking, and 1.14 % (95 % CI 0.95, 1.33) to 6.31 % (95 % CI 5.88, 6.75) for obesity.

### Impact of successive changes to comorbidity algorithm

The impact of each successive change to the algorithm varied by comorbid condition (Fig. 1). When the timeframe was expanded to include diagnoses coded during the surgical admission (algorithm 2), the percentage of women with diabetes, hypertension, anemia, smoking, and obesity increased 19, 37, 26, 319, and 196 %, respectively. The impact of dropping the requirement for spacing of provider/outpatient claims > 30 days apart in algorithm 3 resulted in an additional 5 % increase in diabetes, 8 % for hypertension, and 25 % for anemia. Requiring only a single provider/outpatient claim for smoking and obesity in algorithm 3 resulted in increased prevalence of smoking (70 %) and obesity (88 %) compared to algorithm 2. Finally, the addition of prescription claims in algorithm 4 increased the percentage of women with diabetes by 13 %, hypertension by 14 %, and smoking by 2 % (Fig. 1). Each successive change from one algorithm to the next resulted in a statistically significant increase in the comorbid condition prevalence with two exceptions (algorithm 4 and smoking, algorithm 3 and diabetes).

We conducted a sensitivity analysis to see if the performance of the algorithms to identify comorbid health conditions varied by age (Table 3). We found the greatest overall percent improvement compared to algorithm 1 in the youngest age group (18–47 years) for diabetes, hypertension, and obesity, while improvement was greatest for deficiency anemia and smoking in the middle age group (48–55 years).

**Table 3:** Comparison of prevalence estimates for comorbidities by age tertile and improvement by algorithm

Comorbidity	Algorithm^a^	Age 18–47 years		Age 48–55 years		Age 56–64 years	
		Prevalence, %	Improvement compared to algorithm 1, %	Prevalence, %	Improvement compared to algorithm 1, %	Prevalence, %	Improvement compared to algorithm 1, %
Diabetes	1	1.39		4.56		8.57	
	2	1.92	38.13	5.26	15.35	10.11	17.97
	3	2.09	50.36	5.62	23.25	10.46	22.05
	4	2.64	89.93	6.35	39.25	11.57	35.01
Hypertension	1	5.62		14.14		24.93	
	2	8.45	50.36	19.16	35.50	33.38	33.89
	3	9.24	64.41	20.98	48.37	35.59	42.76
	4	11.04	96.44	24.00	69.73	40.23	61.37
Deficiency anemia	1	4.44		4.35		3.67	
	2	5.47	23.20	5.86	34.71	4.66	19.18
	3	6.77	52.48	7.37	69.43	5.82	48.85
Smoking	1	1.78		1.61		1.94	
	2	7.03	294.94	7.94	393.17	7.41	281.96
	3	11.96	571.91	13.51	739.13	12.55	546.91
	4	12.20	585.39	13.77	755.28	12.70	554.64
Obesity	1	0.84		1.28		1.31	
	2	2.79	232.14	3.49	172.66	3.86	194.66
	3	5.07	503.57	6.20	384.38	7.74	490.84

^a^ See Table 1 for description of each algorithm

In the final algorithm for each comorbid condition (i.e., algorithm 3 for anemia and obesity, algorithm 4 for diabetes, hypertension, and smoking), provider/outpatient facility claims contributed more to the prevalence estimate than inpatient facility claims (Table 4). Prescription drug claims captured more women with diabetes than either inpatient facility or outpatient facility/provider claims alone. For hypertension, prescription claims plus at least a single coded medical claim identified more women than were identified by either a single inpatient facility or ≥ 2 outpatient facility/provider claims. In contrast, smoking cessation prescription claims did not add significantly to the prevalence estimate of smoking (Table 4).

**Table 4:** Prevalence of comorbidities by claims data source in the final algorithm^a^ for 11,973 women

Claim source	Diabetes, n (%)	Hypertension, n (%)	Deficiency anemia, n (%)	Smoking, n (%)	Obesity, n (%)
Inpatient facility	366 (3.06)	1,382 (11.54)	215 (1.80)	746 (6.23)	314 (2.62)
Provider or outpatient facility	637 (5.32)	2,147 (17.93)	642 (5.36)	1,017 (8.49)	540 (4.51)
Prescription drug	641 (5.35)	2,577 (21.52) ^b^	n/a	61 (0.51)	n/a
Inpatient facility, provider, or outpatient facility (algorithm 3)	718 (6.00)	2,603 (21.74)	796 (6.65)	1,515 (12.65)	756 (6.31)
Inpatient facility, provider, outpatient facility, or prescription drug (algorithm 4)	813 (6.79)	2,978 (24.87) ^b^	n/a	1,541 (12.87)	n/a

*n/a* not applicable

^a^ Final algorithm was algorithm 3 for deficiency anemia and obesity and algorithm 4 for diabetes, hypertension, and smoking; see Table 1 for description of each algorithm

^b^ By definition all with Rx had at least one facility or provider medical claim

**Fig. 2:**
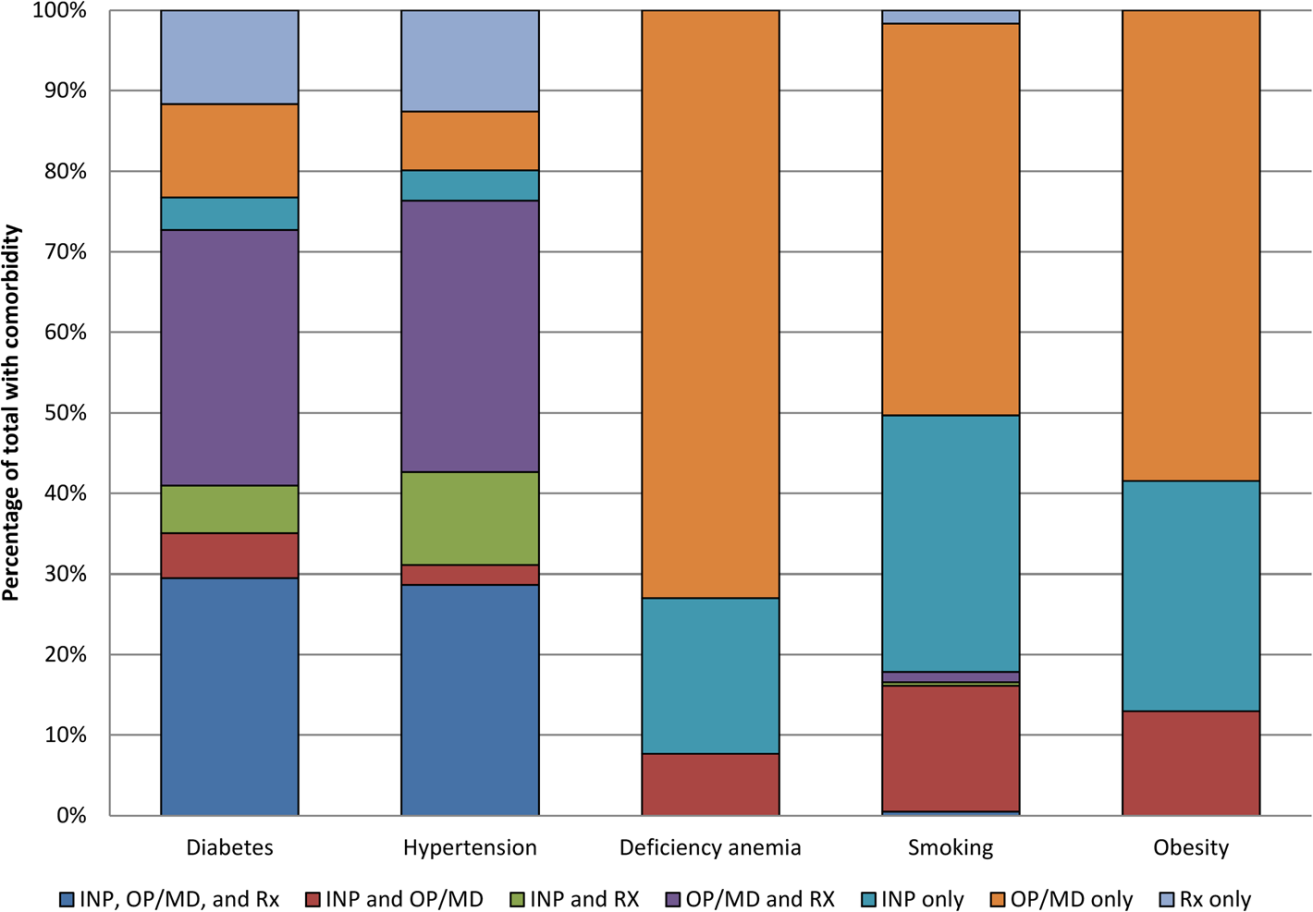
Claim Sources Used to Define Comorbidities in Final Algorithm* Among Those Positive for the Comorbidity. INP = inpatient facility claim, OP/MD = outpatient facility or provider claim, Rx = prescription drug claim. *algorithm 3 for deficiency anemia and obesity, algorithm 4 for diabetes, hypertension, and smoking

We examined the combination of data sources coded for individual conditions and relevant medications (Fig. 2). In algorithm 4, 32 % of women who met the definition for diabetes and 34 % of women with hypertension had both provider/outpatient facility claims and medication claims for the condition. Thirty percent of women with diabetes and 29 % with hypertension were coded positive in all three data sources (i.e., inpatient facility, provider/outpatient facility, and prescription drug). Provider/outpatient facility claims alone were the most common source coded for anemia (73 %), smoking (49 %), and obesity (58 %), followed by coding on inpatient facility claims alone with 19, 32, and 29 % respectively. Only a small percentage had both inpatient facility and provider/outpatient facility claims positive for anemia, smoking, or obesity (8, 16, and 13 %, respectively).

To determine the clustering of coding of diagnoses before mastectomy in this younger population, we analyzed the diagnosis codes present on medical claims in the month prior to mastectomy. In this time period, 64 % of the diagnoses on medical claims related to the diagnosis of breast cancer (invasive or *in situ* cancer, history of breast cancer) or suspicion of cancer (e.g., mammographic abnormality, lump in breast). Invasive or *in situ* cancer codes were particularly common, accounting for 44 % of all diagnoses on medical claims in the month prior to mastectomy.

### Prevalence of comorbid conditions in claims data versus national estimates

We calculated national estimates for the prevalence of diabetes (6.01 %), hypertension (19.84 %), smoking (17.63 %) and obesity (25.51 %) in adult insured women under the age of 65 years using data from the 2007 BRFSS, and used the estimate of iron deficiency anemia (3.67 %) derived from the 2000 NHANES [22]. The revised estimate of diabetes prevalence in our mastectomy population calculated by algorithms 2 (5.70 %; *p* = 0.160) and 3 (6.00 %; *p* = 0.952) and the estimate of hypertension prevalence calculated in algorithm 2 (20.15 %; *p* = 0.402) were not significantly different from the national estimates (Fig. 1).

### Comorbid condition prevalence in claims data by year and compared to medical record review

We compared the prevalence of the five comorbid conditions among women with mastectomy in 2004–2008 versus 2009–2011 to look at the impact of the increase in ICD-9-CM diagnosis code fields in the HIRD^SM^ from 5 to 12 (see Additional file 1). For diabetes, hypertension, smoking, and obesity, the prevalence was significantly higher in 2009–2011 for all 4 algorithms compared to the earlier time period. The prevalence of anemia was higher among women with mastectomy in 2004–2008, although it did not reach statistical significance for algorithm 2.

We abstracted information on current smoking and height and weight to calculate BMI from the medical records of 290 women in the cohort, although not all records contained the specific documentation (75 % had information on smoking and 60 % of records had both weight and height). The specificity of coding for both current and ever-smoking and for obesity was very high (Table 5). The PPV of coding was high for both conditions, although the PPV for coding was lower when smoking was defined as current smoking. Conversely, the sensitivity and NPV of coding for smoking were higher when coded smoking was compared to current smoking status documented in the medical record, rather than ever smoked. For obesity, the specificity and PPV were high and the sensitivity and NPV were low for all 3 algorithms (Table 5). The BMI for obese women who were coded with ICD-9-CM diagnosis codes for obesity in claims was significantly higher than the BMI in obese women with no encounters coded for obesity (median BMI 40.20 vs. 32.85, *p* < 0.001, Mann–Whitney U test).

**Table 5:** Results of chart validation of smoking and obesity compared with insurance claims

	Chart and claims, n	Chart only, n	Claims only, n	Not in chart or claims, n	Sensitivity, %	Specificity, %	PPV, %	NPV, %
Smoking per claims algorithm^a^ versus current smoker in chart^b^								
Algorithm 1	4	20	1	191	16.67	99.48	80.00	90.52
Algorithm 2	12	12	7	185	50.00	96.35	63.16	93.91
Algorithm 3	15	9	14	178	62.50	92.71	51.72	95.19
Algorithm 4	15	9	15	177	62.50	92.19	50.00	95.16
Smoking per claims algorithm^a^ versus current or former smoker in chart^b^								
Algorithm 1	5	62	0	150	7.46	100.00	100.00	70.75
Algorithm 2	18	49	1	149	26.87	99.33	94.74	75.25
Algorithm 3	24	43	5	145	35.82	96.67	82.76	77.13
Algorithm 4	25	42	5	145	37.31	96.67	83.33	77.54
Obesity per claims algorithm^a^ versus BMI ≥ 30 in chart^b^								
Algorithm 1	4	52	0	118	7.14	100.00	100.00	69.41
Algorithm 2	7	49	0	118	12.50	100.00	100.00	70.66
Algorithm 3	10	46	1	117	17.86	99.15	90.91	71.78

*BMI* body mass index, *PPV* positive predictive value, *NPV* negative predictive value

^a^ See Table 1 for description of each algorithm

^b^ Among those with non-missing information in the medical chart (*n* = 216 current smoker, *n* = 217 current or former smoker, *n* = 174 BMI)

## Discussion

We found progressively increasing prevalence of comorbid conditions in a population of younger women who underwent mastectomy by including the surgical admission to identify conditions, eliminating minimum spacing or need for more than one outpatient/provider claims coded for the condition, and identifying conditions with prescription claims, where possible. For all comorbid conditions, each change to the algorithm resulted in additional women detected with the underlying health condition and prevalence values closer to the national estimates for insured adult women, particularly for the younger women in our population. These five comorbid conditions served as illustrative examples of the range of opportunities to increase capture of comorbid conditions depending on the specific characteristics of the condition.

The inclusion of diagnosis codes from the mastectomy admission resulted in substantially increased prevalence of smoking, obesity, and hypertension. This finding is in agreement with previous studies which found the addition of diagnosis codes from the index admission was important to more accurately capture underlying comorbidities [10, 12, 15]. One explanation for this increase in prevalence could be the relevance of these conditions at the time of surgery. In the month prior to mastectomy in this younger population, 64 % of the diagnoses coded on medical claims concerned the acute diagnosis (e.g., breast cancer, mammographic abnormality). Diagnoses of comorbid conditions may not have been considered relevant until the inpatient surgical admission, when hypertension, obesity, and smoking are important considerations in the administration of anesthesia and operative care. The inclusion of diagnoses from the mastectomy admission is justified for the comorbid conditions we chose to analyze since they are unlikely to arise during the mastectomy hospitalization.

There was a small but significant increase in the prevalence of hypertension and anemia when any two coded provider/outpatient facility claims were considered evidence for the condition, dropping the requirement for coding during encounters spaced at least 30 days apart. This may also be due to the younger mastectomy population we studied, in which healthcare encounters for workup and treatment are clustered during a relatively short time interval. We are unaware of any other studies that have assessed the spacing of outpatient claims. There was a large increase in prevalence of smoking and obesity when only one provider/outpatient facility claim was required to establish the diagnosis. The requirement of ≥ 2 outpatient/provider claims to identify a condition was incorporated in comorbidity algorithms to account for inaccuracy in coding on outpatient claims [13, 24], and the lack of specific codes for diagnostic workup. Since no testing is required to establish the diagnosis of obesity or smoking, we removed the requirement for coding during multiple encounters, resulting in an 88 and 70 % increase in the prevalence of obesity and smoking, respectively. The resulting increase put the calculated 12.65 % smoking prevalence in our privately insured population much closer to the 2007 national estimate of current smoking in non-elderly adult women of 17.63 %.

Finally, the addition of outpatient prescription claims significantly increased the calculated prevalence of diabetes and hypertension, and in fact prescription claims identified more women with diabetes than medical claims. Medications have been used to identify chronic conditions alone [25–28], or in combination with diagnosis codes on medical claims [29, 30]. Adding medications to a medical claims algorithm improved identification of osteoporosis in a Canadian study [31, 32]. In contrast, prescription claims for smoking deterrents were uncommon and did not add to the prevalence estimate for smoking. Other investigators have used outpatient pharmacy data to estimate the prevalence of several chronic conditions, and found good agreement with health survey data for chronic conditions treated with specific agents, including diabetes and thyroid disorders [33]. The estimated prevalence of chronic conditions based on prescription drug utilization was higher than national estimates based on survey data in some studies [33, 34].

We found that the calculated prevalence for diabetes and hypertension in the mastectomy population were higher than prevalence estimates for women aged 18–64 years using the BRFSS and NHANES data. This could be due to the association of diabetes and hypertension with increased incidence of breast cancer [35–37]. Our calculated prevalence of anemia, which included iron deficiency and other anemias, may be higher than the national estimate since the NHANES testing was limited to iron deficiency anemia. In addition, the prevalence of anemia may be higher in the mastectomy population since some women may have developed anemia secondary to neoadjuvant chemotherapy, which could have been coded using a diagnosis code in our algorithm for “deficiency” anemia.

In contrast, our calculated prevalence of obesity was much lower than the estimated national prevalence of 26 % in non-elderly adult women, likely due to the low sensitivity (18 %) of ICD-9-CM diagnosis codes to identify obesity that we found in medical record review. The very low sensitivity and high PPV of ICD-9-CM diagnosis codes to identify obesity in our study has also been reported for ICD-10 codes in Canada [38]. In our medical record validation we found that the median BMI for obese women with an ICD-9-CM diagnosis code for obesity was higher than the BMI of obese women who were not coded for obesity in the prior year, suggesting that morbid obesity is more likely to be coded than obesity per se. Supporting this is the similarity of the 6.31 % prevalence of coded obesity in our mastectomy population to the 8.29 % prevalence of morbid obesity in the NPCR Breast Cancer study [39].

Similarly, the prevalence of smoking in the mastectomy population defined by one or more encounters coded for tobacco use disorder was 12.87 %, lower than the 2007 national estimate of 17.63 % from the BRFSS. Our lower prevalence may be due to the relatively low sensitivity (62.50 %) of coding for smoking we found compared to current smoking documented in the medical records. Coding of tobacco history or disorder had higher sensitivity to identify current smokers than ever smokers (62.50 % vs. 37.31 %, respectively), but the PPV was lower for current compared to ever-smoking. These results suggest that tobacco use coding has relatively high specificity, but may not distinguish between current and former smokers.

For all comorbid conditions other than anemia, the prevalence estimates were higher when 12 diagnosis code fields were available in the later time period of the database rather than 5 fields. The trends of increasing prevalence with sequential changes in our coding algorithm were the same in both time periods. The reason for the lower prevalence of anemia in the later years of the study (2009–2011) may be due to the addition of a specific diagnosis code for antineoplastic chemotherapy-induced anemia in 2009, which is not included in the standard comorbidity category of anemia.

We found greatest improvement in our final algorithm in the younger third of the population compared with the older women in the cohort for diabetes, hypertension, and obesity. We hypothesized that comorbidities may not be captured as well among younger women with existing algorithms, owing to their different patterns of healthcare utilization compared to older women, which proved true for these three comorbid conditions. While improvements in capture of the five conditions we studied were evident in the older age groups as well (albeit to a lesser extent), particularly for anemia and smoking, these women in our cohort may be more similar to the older, sicker populations that were used to develop the traditional comorbidity algorithms [1, 4, 13].

Previous studies have compared claims-based comorbidity measures or modifications based on improvement in ability to predict an outcome, rather than improved accuracy of condition identification. Most investigators have based improvement in comorbidity measures on improvement in model performance (e.g., concordance statistic) to predict mortality [7–10, 12, 14, 29, 40–43], although other outcomes such as healthcare costs [44, 45], readmissions [30], and progression of disease [41] have also been assessed. In contrast, we compared the prevalence of comorbidities calculated using claims data to health condition estimates in adult women of the same age reported in the BRFSS and NHANES survey populations. While we compared survey respondents with similar demographics to our population with respect to age, sex, and insurance status (BRFSS only), these national estimates may still not be completely comparable to the breast cancer population. The prevalence of a given comorbid condition in our commercially insured population may vary by other demographics that we could not account for, such as state of residence and type of insurance. In addition, the national estimates from the BRFSS survey are subject to limitations including self-report and recall bias.

We utilized a population of younger women undergoing mastectomy in order to assess modifications of a comorbidity algorithm in a non-elderly population. The patterns of healthcare utilization in our population of women, the majority of whom were newly diagnosed with breast cancer, may be different from other populations; therefore the generalizability of our findings is unknown. Future studies to determine if our findings hold in other non-elderly surgical and non-surgical populations, in men, and in patients with other types of health insurance are warranted. Men in particular have different patterns of healthcare utilization that may impact the performance of the algorithm.

## Conclusions

We found progressively increasing prevalence of diabetes, hypertension, and anemia using claims data from a large population of non-elderly adult women undergoing mastectomy by altering the standard claims-based comorbidity algorithm to include diagnosis codes assigned during the surgical admission and removal of the requirement for spacing of diagnosis codes in outpatient encounters. Addition of outpatient pharmacy claims resulted in further increases in the calculated prevalence of diabetes and hypertension. We found progressively increasing prevalence of obesity and smoking by including diagnosis codes assigned during the surgical admission and by requiring only one coded inpatient or outpatient encounter, although medical record review to verify coding accuracy revealed low sensitivity of ICD-9-CM diagnosis codes to identify both health conditions. An important strength of our study is the large, longitudinal, population-based sample of younger persons representing hundreds of facilities from different geographic regions and different practice patterns. We assessed improvement in the coding algorithms compared to national survey estimates of the prevalence of the conditions in adult, non-elderly, insured women. Our results suggest a “one size fits all” approach to identifying comorbid conditions in claims data may not be the best way to identify individuals with a particular condition, and that investigators performing studies using claims data in younger, privately insured individuals may want to modify their strategy based on the particular comorbid conditions of interest. Additional research investigating strategies to optimize identification of comorbid health conditions in younger populations is needed to confirm these findings.

## Additional file

Additional file 1: Diagnosis Codes and Drug Categories Used to Identify Comorbidities and Prevalence ofComorbidities Among Women From 2004–2008 vs. 2009–2011. (DOCX 15 kb)

## Abbreviations

BMI: Body Mass Index
BRFSS: Behavioral Risk Factor Surveillance System
CI: Confidence Interval
CPT-4: Current Procedural Terminology, 4^th^ edition
HCPCS: Healthcare Common Procedure Coding System
HIRD^SM^: HealthCore Integrated Research Database
ICD-9-CM: International Classification of Diseases, 9^th^ Revision, Clinical Modification
NHANES: National Health and Nutrition Examination Survey
NPV: Negative Predictive Value
PPV: Positive Predictive Value
UB-04: Uniform Billing Revenue
