# Supplementary material for: Modification of claims-based measures improves identification of comorbidities in non-elderly women undergoing mastectomy for breast cancer: a retrospective cohort study
Source: BMC Health Serv Res. 2016 Aug 16;16:388. doi: 10.1186/s12913-016-1636-7 (PMC4986377; doi:10.1186/s12913-016-1636-7)
Supplement: Additional file 1: — Diagnosis Codes and Drug Categories Used to Identify Comorbidities and Prevalence ofComorbidities Among Women From 2004–2008 vs. 2009–2011. (DOCX 15 kb) [file 12913_2016_1636_MOESM1_ESM.docx]

**Diagnosis Codes and Drug Categories Used to Identify Comorbidities**

| **Comorbidity** | **ICD-9-CM diagnosis code** | **Drug category** |
| --- | --- | --- |
| Diabetes^*^ | 249–249.91, 250–250.93, 648.00–648.04 | Antidiabetics |
| Hypertension | 401.0, 401.1, 401.9, 402.00–405.99, 437.2, 642.00–642.04, 642.10–642.24, 642.70–642.94 | Beta blockers, calcium channel blockers, antihypertensives, diuretics, peripheral vasodilators, phosphodiesterase inhibitors, endothelin receptor antagonists,  prostaglandin vasodilators,  vasoactive natriuretics peptides |
| Deficiency anemia | 280.1–281.9, 285.21–285.29, 285.9 |  |
| Smoking | 305.1, 649.00–649.04, V15.82 | Smoking deterrents |
| Obesity^†^ | 278.00, 278.01, 278.03, 649.10–649.14, 793.91, V85.30–V85.45 |  |

^*^ ICD-9-CM diagnosis code 775.1 (neonatal diabetes mellitus) excluded since not applicable to an adult population.

^†^ ICD-9-CM diagnosis code V85.54 (pediatric body mass index ≥ 95th percentile for age) excluded since not applicable to an adult population.

**Prevalence of Comorbidities Among Women From 2004–2008 vs. 2009–2011**

| **Algorithm** | **Comorbidity** | **Prevalence**  **2004–2008, %**^*^ | **Prevalence**  **2009–2011, %**^*^ | ***P*** |
| --- | --- | --- | --- | --- |
| Algorithm 1 | Diabetes | 4.29 | 5.66 | 0.001 |
|  | Hypertension | 13.67 | 16.63 | <0.001 |
|  | Deficiency anemia | 4.79 | 3.28 | <0.001 |
|  | Smoking | 1.21 | 2.77 | <0.001 |
|  | Obesity | 0.79 | 1.74 | <0.001 |
| Algorithm 2 | Diabetes | 5.22 | 6.55 | 0.003 |
|  | Hypertension | 18.84 | 22.42 | <0.001 |
|  | Deficiency anemia | 5.60 | 4.86 | 0.081 |
|  | Smoking | 5.73 | 10.44 | <0.001 |
|  | Obesity | 2.29 | 5.25 | <0.001 |
| Algorithm 3 | Diabetes | 5.51 | 6.85 | 0.003 |
|  | Hypertension | 20.40 | 24.07 | <0.001 |
|  | Deficiency anemia | 7.06 | 5.93 | 0.017 |
|  | Smoking | 10.19 | 16.95 | <0.001 |
|  | Obesity | 4.61 | 9.28 | <0.001 |
| Algorithm 4 | Diabetes | 6.42 | 7.44 | 0.031 |
|  | Hypertension | 23.83 | 26.68 | 0.001 |
|  | Smoking | 10.44 | 17.11 | <0.001 |

^*^ Claims from women with mastectomy in 2004-2008 had 5 ICD-9-CM diagnosis code fields, women with mastectomy from 2009–2011 had claims with 12 ICD-9-CM diagnosis code fields.
